# Tailoring dual antiplatelet therapy for stroke prevention: a meta-analysis of timing, duration, regimen, and stroke subtypes

**DOI:** 10.3389/fphar.2025.1516402

**Published:** 2025-04-24

**Authors:** Naif M. Alhawiti, Sherouk Fouda, Naser D. Alotaibi, Hassan A. Madkhali, Fahad K. Alsharef, Jamal M. Alhawiti, Ashraf El-Metwally

**Affiliations:** ^1^ Department of Clinical Laboratory Sciences, College of Applied Medical Sciences, King Saud bin Abdulaziz University for Health Sciences, Riyadh, Saudi Arabia; ^2^ King Abdullah International Medical Research Center, Riyadh, Saudi Arabia; ^3^ Department of Biomedical Sciences, School of Health and Biomedical Sciences, RMIT University, Melbourne, VIC, Australia; ^4^ College of Medicine, King Saud Bin Abdulaziz University for Health Sciences, Riyadh, Saudi Arabia; ^5^ Division of Neurology, Department of Medicine, King Abdulaziz Medical City, National Guard Health Affairs, Riyadh, Saudi Arabia; ^6^ Department of Pharmacology and Toxicology, College of Pharmacy, Prince Sattam Bin Abdulaziz University, Al-Kharj, Saudi Arabia; ^7^ College of Medicine, Aljouf University, Aljouf, Saudi Arabia; ^8^ College of Public Health and Health Informatics, King Saud bin Abdulaziz University for Health Sciences, Riyadh, Saudi Arabia

**Keywords:** dual antiplatelet therapy, efficacy, safety, recurrent ischemic stroke, systematic review and meta-analysis

## Abstract

**Background:**

Dual antiplatelet therapy (DAPT) is commonly used for secondary stroke prevention, but the optimal timing and duration of treatment remain uncertain. This meta-analysis investigated the efficacy and safety of DAPT compared to any single antiplatelet therapy in stroke patients. We examined the effectiveness of DAPT versus monotherapy, stratified by stroke type, timing of intervention onset, and duration of DAPT.

**Methods:**

We systematically searched electronic databases for randomized controlled trials (RCTs) comparing DAPT with any single antiplatelet therapy in stroke patients. Data from 30 RCTs involving 75,504 patients were pooled using a random-effects model. The key outcomes were recurrent ischemic stroke, major adverse cardiovascular events (MACE), and major bleeding. Additional studied outcomes included hemorrhagic stroke and mortality. Subgroup analysis examined the effectiveness of DAPT versus any single antiplatelet therapy, stratified by stroke type (ischemic stroke, lacunar stroke, and TIA or ischemic stroke), timing of intervention onset (within 12 h, 24 h, 48 h, 72 h, and 7–180 days), duration of DAPT (short-term: up to 30 days; long-term: beyond 30 days) and DAPT regimens (Aspirin and Clopidogrel, Aspirin and Cilostazol, Aspirin and Dipyridamole, and Clopidogrel and Cilostazol, etc.).

**Results:**

DAPT significantly reduced recurrent ischemic stroke (RR 0.69, 95% CI 0.60–0.79) and MACE (RR 0.77, 95% CI 0.69–0.87), but did not significantly affect hemorrhagic stroke (RR 1.28, 95% CI 0.80–2.07), major bleeding (RR 1.10, 95% CI 0.91–1.33), or mortality (RR 1.01, 95% CI 0.88–1.15). Subgroup analyses showed that aspirin plus clopidogrel reduced recurrent stroke (RR 0.69, 95% CI 0.59–0.82) and MACE (RR 0.82, 95% CI 0.75–0.91). Early DAPT initiation (within 12–24 h) significantly reduced recurrent ischemic stroke (RR 0.73, 95% CI 0.57–0.92 and RR 0.66, 95% CI 0.52–0.84, respectively) and MACE (RR 0.78, 95% CI 0.62–0.98 and RR 0.81, 95% CI 0.72–0.93, respectively), but increased major bleeding (RR 2.32, 95% CI 1.10–4.86 and RR 1.34, 95% CI 1.20–1.49, respectively). Short-term DAPT (≤30 days) showed a greater reduction in recurrent ischemic events (RR 0.65, 95% CI 0.53–0.79) than long-term DAPT (>30 days; RR 0.72, 95% CI 0.60–0.86).

**Conclusion:**

DAPT effectively reduces recurrent ischemic stroke and MACE, especially when initiated within 12–24 h using aspirin plus clopidogrel. Short-term DAPT (≤30 days) may be optimal for recurrent stroke prevention. Clinicians should carefully weigh benefits and risks when personalizing DAPT strategies.

## Introduction

Individuals who have been diagnosed with a transient ischemic attack or small stroke are more likely to have repeated thrombotic episodes, especially in the 3 months that follow (Amarenco et al., 2016; Timp et al., 2019). The evidence from randomized controlled trials (RCTs) and subsequent meta-analyses have shown the efficacy and effectiveness of mono antiplatelet therapy such as aspirin or clopidogrel among patients with minor ischemic strokes or transient ischemic attacks (Collaboration, 2002; Xian et al., 2016). Antiplatelet therapy is a well-established pharmaceutical regime for reducing the probability of thrombotic events among patients diagnosed with ischemic stroke (Rothwell et al., 2007). The likely advantages of aspirin in alleviating the burden and reducing the severity of recurrent stroke are well documented in the scientific literature (Author anonymous, 1997; Rothwell et al., 2016). Pharmaceutical regimes, such as dipyridamole alone or in combination with aspirin, is not found to be superior in inhibiting the activity of platelets to mono therapy with aspirin (Rothwell et al., 2016). In addition, triple pharmaceutical regimen with antiplatelet drugs, such as combination of aspirin, clopidogrel, and dipyridamole increases the hazards of major bleeding among patients diagnosed with stroke (Bath et al., 2018; van Rein et al., 2019).

Since the antiplatelet drugs such as aspirin or clopidogrel do not antagonize the same steps in the platelet activation process, there is rationale to anticipate that combination of two antiplatelet agents may enhance antithrombotic efficacy (Furie et al., 2011). Hence, clinicians and experts in the field of cardiovascular diseases have shifted their focus in examining the role of dual antiplatelet therapy (DAPT) (e.g., aspirin and clopidogrel, aspirin and ticagrelor, aspirin and cilostazol, and aspirin and dipyridamole) in decreasing the prevalence and burden of recurrent ischemic strokes and associated comorbidities. Additionally, the emerging scientific evidence suggests the beneficial effect of dual antiplatelet therapy in reducing the incidence of recurrent ischemic stroke and other cardiovascular events among patients diagnosed with ischemic stroke. While the DAPT such as combination of aspirin and clopidogrel has been found successful among patients with acute coronary syndromes (Cuisset et al., 2017), therapeutic effect of the dual therapy among patients with ischemic stroke is not well studied. Therefore, it is important to determine whether these dual regimens can be considered appropriate alternatives to standard monotherapeutic antiplatelet regimens for the subsequent events of a stroke. Besides, while DAPT may provide additional protection against recurrence of stroke, the combination of two antiplatelet therapy may increase the risk of adverse events and complications. Thus, it would be helpful to assess the therapeutic efficacy and safety of DAPT by synthesizing the evidence from existing randomized controlled trials that have examined both efficacy and safety of combination of two antiplatelet therapies. Hence, we addressed this gap in knowledge by conducting a systematic review and meta-analysis of the randomized controlled trials examining the efficacy and safety of dual antiplatelet therapy among stroke patients. Additionally, in this comprehensive meta-analysis, we present a novel approach to evaluating the effectiveness of DAPT by incorporating an extensive subgroup analysis and including a broader range of studies. Our meta-analysis differentiates itself from previous research by stratifying data based on stroke type, timing of intervention onset, and duration of DAPT, areas previously unexplored in literature. This innovative methodology allows for a more nuanced understanding of DAPT’s efficacy across various clinical contexts. By integrating a wider array of studies and conducting detailed subgroup analyses, our meta-analysis offers valuable insights into optimizing DAPT regimens for preventing recurrent ischemic events, ultimately enhancing the precision and applicability of our findings to clinical practice. The findings of this review will help clinicians and policymakers to make informed evidence-based decisions for patients diagnosed with stroke.

## Materials and methods

### Data bases and search terms

We undertook the systematic review and meta-analysis using PRISMA (Preferred reporting items for Systematic reviews and meta-analysis) guidelines (Page et al., 2021). A comprehensive literature search was conducted to identify all relevant RCTs comparing DAPT with single antiplatelet therapy in stroke patients. The search encompassed three electronic databases: PubMed, Web of Science, and EMBASE, and spanned the period from January 2000 to December 2024 to include the most recent literature. The search strategy employed a combination of keywords and controlled vocabulary terms related to the population, intervention, comparison, and outcomes of interest (Supplementary Table S1). Specifically, terms related to stroke (including “stroke,” “ischemic stroke,” “cerebral infarction,” “cerebral ischemia,” and “transient ischemic attack”) were combined using Boolean operator “OR.” These population terms were then combined using “AND” with terms related to DAPT (“dual antiplatelet therapy,” “combined antiplatelet treatment,” “dual *versus* monoantiplatelet”). Similarly, terms related to single antiplatelet therapy (“single antiplatelet agent,” “antiplatelet agent”) were searched using “OR” and then combined with the population terms using “AND.” Individual searches for specific antiplatelet agents (aspirin, clopidogrel, ticagrelor, dipyridamole, and cilostazol) were also performed and combined with the population terms using “AND” to ensure comprehensive capture of relevant studies regardless of specific DAPT combinations. All searches were limited to studies involving adult populations (including young adult, middle aged, aged, and very elderly) and published between 2000 and December 2024. To identify relevant study designs, the term “randomized controlled trial” was included in the search strategy.

### Eligibility criteria

We searched the databases using PICO framework, that helped us to apply the eligibility criteria using major concepts such as Population, Intervention, Comparison, and Outcome (Amir-Behghadami and Janati, 2020). PICO framework guided the development of our eligibility criteria, focusing on Population, Intervention, Comparison, and Outcome (Amir-Behghadami and Janati, 2020). We included randomized controlled trials (RCTs) involving patients with acute stroke, encompassing all subtypes (ischemic, hemorrhagic) and transient ischemic attacks (TIAs). The intervention of interest was dual antiplatelet therapy (DAPT), defined as the combination of two antiplatelet agents. This included, but was not limited to, the combination of aspirin with clopidogrel or ticagrelor. Other DAPT combinations, such as aspirin plus dipyridamole, were also eligible if evaluated in the included RCTs. The comparison group was single antiplatelet therapy. To avoid ambiguity, single antiplatelet therapy was defined as any antiplatelet medication administered as monotherapy (e.g., aspirin, clopidogrel, ticagrelor, cilostazol, dipyridamole). This definition clarifies that comparisons of DAPT to interventions other than single antiplatelet agents (such as thrombolysis or combination therapies involving non-antiplatelet drugs) were not eligible. The main outcomes were the occurrence of recurrent ischemic stroke during the follow-up period reported in each trial, major adverse cardiovascular events (MACE) (as specified by authors in their studies), and major bleeding (defined according to the criteria used in the original trials and details are provided in Supplementary Table S2). Other outcomes studied were all-cause mortality and hemorrhagic stroke. Only completed and peer-reviewed RCTs were eligible. Conference abstracts and other non-peer-reviewed materials were excluded to ensure the quality and reliability of the evidence, as abstracts often lack the detailed information necessary for rigorous assessment and data extraction. Studies were included only if they provided sufficient data to calculate pooled risk ratios (RRs) or hazard ratios (HRs), including the number of events and the total number of participants in each group. Studies were excluded if they: 1) evaluated interventions other than DAPT as defined above; 2) did not employ an RCT design; 3) were qualitative studies, reviews, editorials, or commentaries; 4) did not provide sufficient data for calculating effect measures; or 5) were published only as abstracts due to limited information.

### Screening and data extraction

At the beginning, titles and abstracts of the articles were screened. This was followed by screening the full texts of the RCTs that were considered eligible based on the above-mentioned criteria. The data from eligible RCTs were extracted using the pre-defined data extraction form. We extracted data on study’s authors, year of publication, study setting or country where study was conducted, intervention, control, outcome, assessment tools to measure outcome, and key findings of the RCT. In addition, we also extracted raw data on the number of patients experiencing recurrent strokes in intervention and control arms along with the sample size of the randomized groups. This data helped us to calculate risk ration and 95% CIs in the meta-analysis.

### Intervention and outcome under investigation

Primary intervention for this review and meta-analysis was dual antiplatelet therapy consisting of aspirin and clopidogrel or aspirin and ticagrelor. We studied the recurrence of ischemic stroke, MACE such as myocardial infarction and mortality due to MACE, and major bleeding that required blood transfusion or lead to compromised hemodynamic response or hypovolemic shock.

## Statistical methods

### Primary analysis

We performed the meta-analysis using random effect models proposed by Der Simonian and Laird, which allowed us to account for heterogeneity across studies. We pooled the results of individual RCTs and computed pooled summary measure-risk ratios (RRs) with 95% CIs. The weights to the individual RCTs were applied based on the sample size and number of events accrued in each group. We generated forest plots that helped visualize the overall pooled effect of DAPT on the primary and secondary outcomes defined in the methods. The RR in the forest plots represents the ratio of the risk of the outcome in the DAPT group compared to the risk in single antiplatelet therapy. An RR less than 1 indicates that DAPT is associated with a lower risk of the outcome. The 95% CI provides a range within which the true RR is likely to lie. Heterogeneity across RCTs was assessed using Q-test and I^2^ statistics. I^2^ value is reported in percentage and provides percentage of heterogeneity not explained by chance alone. Low, moderate, and high heterogeneity was suggested by 25%–50%, 50%–75%, >75%, respectively. In addition, we assessed publication bias using funnel plot and Egger’s regression test of asymmetry. Since the Egger’s test on its own is not powerful enough to detect publication bias, we visualized funnels plots and ran Egger’s regression test. We performed the analysis using R 4.2.2.

### Subgroup analysis

In this study, subgroup analyses were conducted to evaluate the effectiveness of DAPT compared to monotherapy for most important outcomes including recurrent ischemic stroke, MACE, and major bleeding. These subgroup analyses were performed by stroke type (ischemic stroke, lacunar stroke, and TIA or ischemic stroke), timing of intervention onset (within 12 h, 24 h, 48 h, 72 h, and 7–180 days), duration of DAPT (short-term: up to 30 days; long-term: beyond 30 days), and DAPT regimens (Aspirin and Clopidogrel, Aspirin and Cilostazol, Aspirin and Dipyridamole, and Clopidogrel and Cilostazol, etc.). These analyses were performed to explore potential variations in treatment effects based on the underlying stroke etiology, the timing of therapy initiation, duration of treatment, and combination of DAPT regimen. For the subgroup analysis we estimated pooled RRs with 95% CIs. Heterogeneity was assessed using the I^2^ statistics, and subgroup differences were tested to determine if these factors influenced the treatment effect.

## Study results

### Search strategy

**FIGURE 1:**
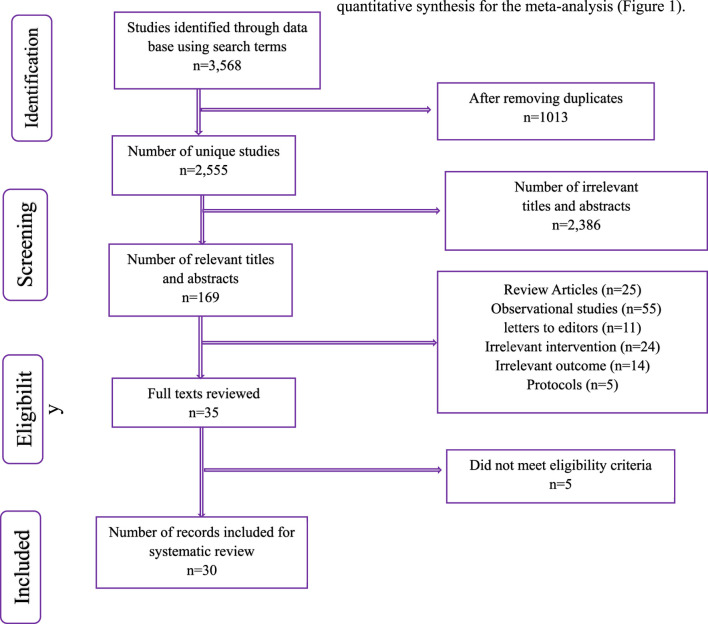
PRISMA Flow chart summarizing the identification and selection of records and final number of RCTs included in the quantitative synthesis for meta-analysis.

A systematic search of electronic databases was conducted to identify relevant studies for inclusion in this meta-analysis. The initial search yielded 3,568 records. After removing 1,013 duplicates, 2,555 unique studies remained. Titles and abstracts were screened, resulting in the exclusion of 2,386 irrelevant records. The remaining 169 potentially eligible studies were further assessed. Of these, 25 were review articles, 55 were observational studies, 11 were letters to the editor, 24 had irrelevant interventions, 14 had irrelevant outcomes, and 5 were study protocols. Following this screening process, 35 full-text articles were retrieved and assessed for eligibility. Five studies were excluded at this stage as they did not meet the pre-defined eligibility criteria. Finally, 30 studies met all eligibility criteria and were included in the systematic review and quantitative synthesis for the meta-analysis (Figure 1).

### Characteristics of eligible studies

Table 1 summarizes the characteristics of various randomized controlled trials (RCTs) included in the systematic review and meta-analysis focusing on dual antiplatelet therapy among stroke patients (n = 30 RCTs; 75,504 patients). The sample size of RCTs ranged between 76 and 15, 604 and the total number of participants across all trials was considerable, amounting to 75,504, providing substantial statistical power for the meta-analysis. These RCTs were published between 2000 and 2024. Out of 30 RCTs, 11 RCTs were multi-country, 7 were conducted in China, 6 in Japan, 3 in Korea, and two in UK, and one was undertaken in North America, reflecting a global effort to address stroke prevention. The average age of participants varied between studies, generally falling in the 60 s (58–70 years), while the gender distribution showed a higher percentage of male participants in most studies. Blinding methods varied, with most studies employing double-blinded or open-label outcome researcher designs. Analysis types were primarily intention-to-treat (n = 19), with eight studies using per protocol analysis and three studies did not report any method of analysis. The rate of participants lost to follow-up also varied, with 11 studies reporting less than 1% while 9 studies reported higher percentages of at least 5% (Table 1).

**TABLE 1:** Characteristics of the RCTs included in systematic review and meta-analysis of Dual antiplatelet therapy among stroke patients (n = 30).

Study	Trial name	Year	Country	n	Age	Gender (%)	Blinding	Analysis type	Lost to follow up
Diener et al. (2004)	MATCH	2004	Multi-country	7,599	66	M: 63 F: 37	Double blinded	Intention to treat	4%
Markus et al. (2005)	CARES	2005	United Kingdom	107	65	M: 69 F: 31	Double blinded	Intention to treat	0
Kwon et al. (2005)	TOSS	2005	Korea	135	62	M: 74 F: 26	Double blinded	NR	25%
Halkes et al. (2006)	ESPRIT	2006	Multi-country	2,739	63	M: 66 F: 34	Open Label: Outcome researcher	Intention to treat	3.80%
Bhatt et al. (2006)	CHARISMA	2006	Multi-country	15,604	64	M: 70 F: 30	Double blinded	Intention to treat	0.50%
Kennedy et al. (2007)	FASTER	2007	North America	392	68	M: 53 F: 47	Double blinded	Intention to treat	1.80%
Geraghty et al. (2010)	EXPRESS	2010	United Kingdom	633	NR	NR	NR	NR	NR
Bath et al. (2010)	PROFESS	2010	Multi-country	1,360	66	M: 64 F: 36	Double blinded	Intention to treat	0.90%
Wong et al. (2010)	CLAIR	2010	Multi-country	98	58	M: 78 F: 22	Open Label: Outcome researcher	Intention to treat	1.00%
Dengler et al. (2010)	EARLY	2010	Multi-country	543	69	M: 62 F: 38	Open Label: Outcome researcher	Per-protocol	2.90%
Hankey et al. (2011) (Dengler et al., 2010)	CHARISMA	2011	Multi-country	4,317	64	M: 63 F: 37	Double blinded	Intention to treat	0.50%
Nakamura et al. (2012)	NA	2011	Japan	76	67	M: 71 F: 29	NR	Per-protocol	6.6% at 1 week and 37% at 6 months
Uchiyama et al. (2011)	JASAP	2011	Japan	1,294	66	M: 71 F: 29	Double blinded	NR	0.2%
Investigators et al. (2012)	SPS3	2012	Multi-country	3,020	63	M: 63 F: 37	Double blinded	Intention to treat	2%
Wang and Zhao (2013)	CHANCE	2013	China	5,170	62	M: 66 F: 34	Double blinded	Intention to treat	0.70%
Han et al. (2013)	ECLIPSE	2013	Korea	203	65.1	M: 75 F: 25	Double blinded	Per-protocol	4.0%
Yi et al. (2014)	NA	2014	China	570	69	M: 55 F: 45	Double blinded	Per-protocol	0.7%
He et al. (2015)	NA	2015	China	647	63	M: 59 F: 41	Single blinded	Per-protocol	6%
Wang et al. (2015)	CHANCE	2015	China	574	70	M: 55 F: 45	Double blinded	Intention to treat	0.70%
Uchiyama et al. (2015)	CATHARSIS	2015	Japan	163	68.3	M: 53 F: 47	Not blinded	Intention to treat	6.70%
Hong et al. (2016)	COMPRESS	2016	Republic of Korea	334	68	M: 66 F: 34	Double blinded	Per-protocol	2.50%
Zuo et al. (2017)	NA	2017	China	200	62	M: 61 F: 39	NR	Per-protocol	7.40%
Johnston et al. (2018)	POINT	2018	Multi-country	4,881	65	M: 55 F: 45	Double blinded	Intention to treat	6.60%
Aoki et al. (2019)	NA	2019	Japan	1,201	69	M: 66 F: 34	Open Label: None	Per-protocol	1.4% at 2 weeks and 7.7% at 3 months
Toyoda et al. (2019)	CSPS.com Trial	2019	Japan	1884	69.5	M: 69 F: 31	Open Label: None	Intention to treat	7.00%
Johnston et al. (2020)	THALES	2020	Multi-country	11,016	65	M: 62 F: 38	Double blinded	Intention to treat	0.50%
Gao et al. (2023)	INSPIRES	2023	China	6,100	65	M: 65 F: 35	Double blinded	Intention to treat	0.10%
Nishiyama et al. (2023)	CSPS.com Trial	2023	Japan	925	69.5	M: 69 F: 31	Open Label: None	Intention to treat	7.00%
Chen et al. (2024)	ATAMIS	2024	China	3,000	66	M: 65 F: 35	Open label: Blinded end point	Modified intention-to-treat	2.80%

M, male; F, female; NA, not applicable; NR, not reported.

### Intervention and outcome details of included RCTs

Table 2 provides intervention and outcome details of the 30 included RCTs included in the review and meta-analysis focusing on dual antiplatelet therapy among stroke patients. All trials compared dual antiplatelet therapy (DAPT) to single antiplatelet therapy. The specific DAPT regimens varied, with the most common combination being aspirin plus clopidogrel (n = 18). Other DAPT regimens included aspirin plus dipyridamole (n = 4), aspirin plus cilostazol (n = 7), and aspirin and ticagrelor (n = 1). The comparator therapy in most trials was aspirin monotherapy (n = 24); however, some studies utilized clopidogrel or monotherapy (n = 4). Intervention duration also varied, with studies employing both short-term DAPT (ranging from 7 days to 30 days) and long-term DAPT (ranging from 1 month to 3.5 years). The timing of DAPT initiation post-stroke also showed variation, with some studies initiating treatment within 12–24 h (n = 11), while 5 RCTs initiated treatment within 48 h, and 8 RCTs started treatment within 72 h or beyond; however, 6 RCTs did not report the timing of onset. With respect to the type of stroke, 10 RCTs included participants with ischemic stroke, 3 RCTs had participants with lacunar stroke, and remaining included patients with ischemic stroke or transient ischemic attack (n = 17). The outcomes assessed across the trials encompassed a range of endpoints related to stroke recurrence, myocardial infarction, major cardiovascular events, death, and bleeding complications as shown in Table 2.

**TABLE 2:** Intervention and outcome details of included RCTs (n = 30).

Study	Trial name	Intervention	Control	Dose	Intervention duration	Duration (binary)	Intervention onset	Stroke type	Outcomes
Diener et al. (2004)	MATCH	Aspirin and Clopidogrel	Clopidogrel	Aspirin:75 mg/day Clopidogrel: 75 mg/day	18 months	Long-term	Within 72 h	TIA or Ischemic stroke	Composite of ischemic stroke, myocardial infarction, major cardiovascular events, and death
Markus et al. (2005)	CARES	Aspirin and Clopidogrel	Aspirin	Aspirin: 75 mg/day Clopidogrel: Loading dose of 300 mg; maintenance dose: 75 mg/day	7 days	Short-term	Within 72 h	TIA or Ischemic stroke (Minor)	TIA, Ischemic strokes, and cerebral hemorrhage
Kwon et al. (2005)	TOSS	Aspirin and cilostazol	Aspirin	Aspirin: 100 mg/day Cilostazol: 200 mg BID	6 months	Long-term	NR	Ischemic stroke	Recurrent stroke, cardiovascular events, and death
Halkes et al. (2006)	ESPRIT	Aspirin and dipyridamole	Aspirin	Aspirin: 30–325 mg Dipyridamole: 200 mg BD	3.5 years	Long-term	Within 72 h	TIA or Ischemic stroke	Death and all major ischemic events (not fatal ischemic stroke, non-fatal myocardial infarction) and major bleeding
Bhatt et al. (2006)	CHARISMA	Aspirin and Clopidogrel	Aspirin	Aspirin: 75–162 mg Clopidogrel: 75 mg/day	2.3 years	Long-term	Within 24 h	TIA or Ischemic stroke	Myocardial infarction, Ischemic stroke, hospitalization, TIA, and death
Kennedy et al. (2007)	FASTER	Aspirin and Clopidogrel	Aspirin	Aspirin: Loading dose of 162 mg followed by maintenance dose of 81 mg/day Clopidogrel: Loading dose of 300 mg followed by maintenance dose of 75 mg/day	3 months	Long-term	Within 24 h	TIA or Ischemic stroke (Minor)	Stroke, TIA, Myocardial infarction, and Death
Geraghty et al. (2010)	EXPRESS	Aspirin and Clopidogrel	Aspirin	Aspirin: 75–300 mg Clopidogrel: 75–300 mg	1–3 months	Long-term	NR	TIA or Ischemic stroke (Minor)	Recurrent ischemic stroke, bleeding
Bath et al. (2010)	PROFESS	Aspirin and dipyridamole	Clopidogrel	Aspirin:25 mg/day Dipyridamole: 200 mg BD Clopidogrel: 75 mg/day	3 months	Long-term	Within 24 h	Acute Ischemic stroke	Recurrent Stroke (Any Type), Death, Myocardial infarction
Wong et al. (2010)	CLAIR	Aspirin and Clopidogrel	Aspirin	Aspirin:75–160 mg/day Clopidogrel: 300 mg loading then 75 mg/day	7 days	Short-term	Within 72 h	TIA or Ischemic stroke (Minor)	Coronary syndrome, recurrent stroke, infarction, and embolism
Dengler et al., 2010	EARLY	Aspirin and dipyridamole	Aspirin	Aspirin: 100 mg (7 days) then 25 mg BID Dipyridamole: 200 mg BD	7 days	Short-term	Within 24 h	TIA or Ischemic stroke	Non-fatal stroke, TIA, myocardial infarction, and major bleeding complications, and mortality
Hankey et al. (2011)	CHARISMA	Aspirin and Clopidogrel	Aspirin	Aspirin:75–162 mg/day Clopidogrel: 75 mg/day	5 years	Long-term	Within 24 h	TIA or Ischemic stroke	Stroke and severe bleeding
Nakamura et al. (2012)	NA	Aspirin and cilostazol	Aspirin	Aspirin: 300 mg/day Cilostazol:100 mg BID	6 months	Long-term	Within 48 h	Ischemic stroke (Minor)	Recurrent stroke, bleeding, and major cardiovascular events
Uchiyama et al. (2011)	JASAP	Aspirin and dipyridamole	Aspirin	Aspirin: 81 mg Dipyridamole: 200 mg BD	1 year	Long-term	NR	Ischemic stroke	Recurrent stroke
Investigators et al. (2012)	SPS3	Aspirin and Clopidogrel	Aspirin	Aspirin: 325 mg/day Clopidogrel: 75 mg/day	3.4 years	Long-term	NR	Lacunar stroke	Recurrent ischemic stroke, intracranial hemorrhage, acute myocardial infarction, and death
Wang and Zhao (2013)	CHANCE	Aspirin and Clopidogrel	Aspirin	Aspirin: 75 mg/day Clopidogrel: Loading dose: 300 mg; maintenance dose: 75 mg/day	21 days	Short-term	Within 24 h	TIA or Ischemic stroke	Recurrent ischemic or hemorrhagic stroke, and vascular death
Han et al. (2013)	ECLIPSE	Aspirin and cilostazol	Aspirin	Cilostazol: 100 mg BID Aspirin 100: mg/day	3 months	Long-term	NR	Lacunar stroke	Recurrent stroke
Yi et al. (2014)	NA	Aspirin and Clopidogrel	Aspirin	Aspirin: 200 mg/day (30 days) then 100 mg/day Clopidogrel: 75 mg/day	30 days	Short-term	Within 48 h	Ischemic stroke (Minor/Moderate)	Recurrent ischemic stroke, Myocardial infarction, Deep vein thrombosis
He et al. (2015)	NA	Aspirin and Clopidogrel	Aspirin	Aspirin: 100 mg Clopidogrel: loading dose: 300 mg; maintenance dose: 75 mg	2 weeks	Short-term	Within 72 h	TIA or Ischemic stroke (Minor)	Recurrent stroke stroke in TIA patients
Wang et al. (2015)	CHANCE	Aspirin and Clopidogrel	Aspirin	Aspirin: 200 mg/day Clopidogrel: 75 mg/day	30 days	Short-term	Within 24 h	Acute large artery atherosclerosis stroke	Recurrent ischemic stroke, major cardiovascular events, and death
Uchiyama et al. (2015)	CATHARSIS	Aspirin and cilostazol	Aspirin	Aspirin: 100 mg/day Cilostazol: 200 mg/day	2 years	Long-term	NR	Ischemic stroke (Major)	Ischemic stroke, major cardiovascular events, and death
Hong et al. (2016)	COMPRESS	Aspirin and Clopidogrel	Aspirin	Aspirin: 300 mg loading, then 100 mg Clopidogrel: 75 mg	30 days	Short-term	Within 48 h	Ischemic stroke (Minor)	Recurrent stroke, myocardial infarction, and vascular death
Zuo et al. (2017)	NA	Aspirin and Clopidogrel	Aspirin	Aspirin: 100 mg Clopidogrel: 50 or 75 mg	3 months	Long-term	Within 24 h	TIA or Acute Ischemic stroke	Recurrent ischemic or hemorrhagic stroke Mortality, and Bleeding
Johnston et al. (2018)	POINT	Aspirin and Clopidogrel	Aspirin	Aspirin: Loading dose of 162 mg; maintenance dose: 81 mg/day Clopidogrel: Loading dose of 600 mg; maintenance dose: 75 mg/day	3 months	Long-term	Within 12 h	TIA or Ischemic stroke (Minor)	Ischemic and hemorrhagic strokes, myocardial infarction, death, and major cardiovascular events
Aoki et al. (2019)	NA	Aspirin and cilostazol	Aspirin	Aspirin: 80–200 mg/day Cilostazol: 200 mg/day	14 days	Short-term	Within 48 h	Ischemic stroke (Minor)	Recurrent ischemic or hemorrhagic stroke, bleeding, and TIA.
Toyoda et al. (2019)	CSPS.com Trial	Aspirin/clopidogrel and cilostazol	Aspirin/Clobidgogrel	Aspirin: 81 or 100 mg Clopidogrel: 50/75 mg Cilostazol: 100 mg, BID	0.5–3.5 years	Long-term	Between 7 and 180 days	Ischemic stroke (Major)	Recurrent ischemic stroke
Johnston et al. (2020)	THALES	Aspirin and Ticagrelor	Aspirin	Aspirin: Loading dose: 325/300 mg; maintenance dose: 75–100 mg/day then 90 mg BID Ticagrelor: Loading dose: 600 mg; maintenance dose: 75 mg/day	1 month	Short-term	Within 24 h	TIA or Ischemic stroke (Minor)	Recurrent ischemic or hemorrhagic stroke, and mortality
Chen et al. (2024)	ARAMIS	Aspirin and Clopidogrel	Aspirin	Aspirin:100 mg/day Clopidogrel: 75–300 mg/day	3 months	Long-term			Intracerebral hemorrhage, bleeding, stroke
Gao et al. (2023)	INSPIRES	Aspirin and Clopidogrel	Aspirin	Clopidogrel: 75–300 mg Aspirin: 100–300 mg	9 months	Long-term	Within 72 h	TIA or Ischemic stroke (Minor)	Recurrent ischemic or hemorrhagic stroke, myocardial infarction, cardiovascular events, and mortality
Nishiyama et al. (2023)	CSPS.com Trial	Aspirin/clopidogrel and cilostazol	Aspirin/Clobidgogrel	Aspirin monotherapy: 81 or 100 mg Clopidogrel monotherapy: 50 or 75 mg Cilostazol (100 mg, BID)	0.5–3.5 years	Long-term	Between 7 and 180 days	Lacunar stroke	Recurrent ischemic stroke, bleeding hemorrhagic stroke (intracerebral/subarachnoid hemorrhage) composite vascular events (stroke, myocardial infarction, and vascular death), and all vascular events
Chen et al. (2024)	ATAMIS	Aspirin and Clopidogrel	Aspirin	Aspirin:100 mg to 300 mg/day Clopidogrel: 75–300 mg/day	7 days	Short-term	Within 48 h	Acute Ischemic stroke	Stroke (ischemic, hemorrhagic), cardiovascular events, bleeding, and death

TIA, transient ischemic attach; NA, not applicable; NR, not reported.

## Overall pooled effect of DAPT on recurrent ischemic stroke

**FIGURE 2:**
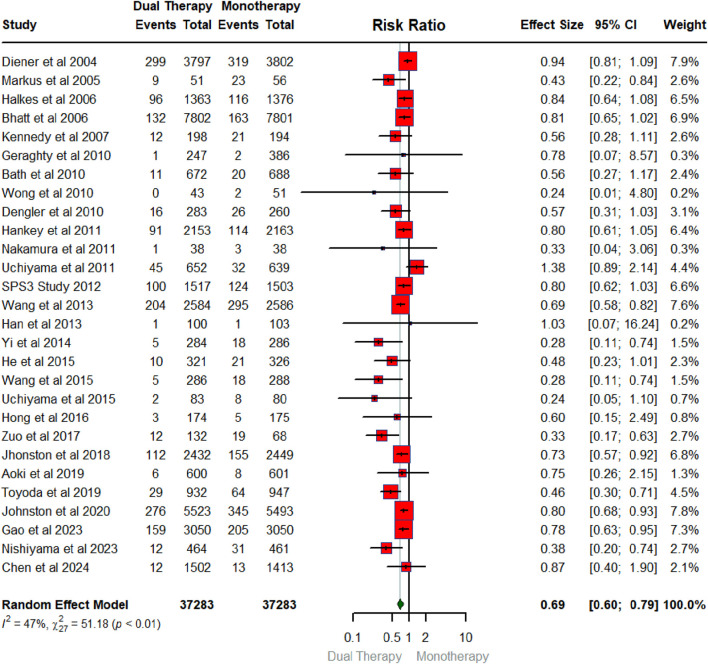
Forest plot illustrating the effect of dual antiplatelet vs. any single antiplatelet therapy in reducing the risk of recurrent ischemic stroke.

Figure 2 below summarizes the findings of a meta-analysis investigating the overall pooled effect of DAPT compared to any single antiplatelet therapy in reducing the risk of recurrent ischemic stroke. The meta-analysis pools data from 28 RCTs reported this primary outcome of recurrent ischemic stroke, encompassing a total of 37,283 participants each in intervention and control arm. For each RCT, the risk ratio (RR) and its associated 95% confidence interval (CI) are shown. The overall pooled RR for the meta-analysis is 0.69 (95% CI: 0.60–0.79). This indicates a statistically significant reduction in recurrent ischemic stroke associated with DAPT compared to monotherapy, as the CI does not cross 1. The heterogeneity among the studies is moderate, as indicated by an I^2^ value of 47%. This suggests some variability in the treatment effect across the trials, although it is not considered substantial.

### Subgroup analysis for recurrent ischemic stroke

#### Subgroup analysis for recurrent ischemic stroke by timing of DAPT initiation

The subgroup analysis by timing of DAPT initiation evaluated the effectiveness of DAPT compared to any single antiplatelet therapy in preventing recurrent ischemic strokes across different timings of initiating DAPT, reported by 23 studies (Supplemental Figure S1). Within 12 h, the pooled RR was 0.73 (95% CI: 0.57–0.92), demonstrating a statistically significant reduction in recurrent ischemic stroke risk favoring DAPT. For the 24-h subgroup, the pooled RR was 0.66 (95% CI: 0.52–0.84), indicating a significant reduction in risk with DAPT, though moderate heterogeneity was observed (I^2^ = 43%). While DAPT initiation within 48 h (RR 0.59, 95% CI 0.30–1.12), and 72 h (RR 0.76, 95% CI 0.57–1.03), and 7–180 days (RR 0.44, 95% CI 0.15–1.25), suggested potential reductions in recurrent ischemic stroke risk, the confidence intervals included 1, indicating uncertainty and statistically non-significant results (Supplementary Figure S1). Moderate heterogeneity was observed across subgroups (I^2^ = 47%), and the test for subgroup differences was significant (p < 0.01), indicating variability in effects across different time periods of commencing DAPT after stroke onset.

#### Subgroup analysis for recurrent ischemic stroke by DAPT duration

Supplementary Figure S2 reveals the findings of subgroup analysis (n = 28) where we compared the effectiveness of DAPT versus any single antiplatelet therapy in preventing recurrent ischemic stroke, stratified by the duration of intervention: short-term (up to 30 days) and long-term (DAPT beyond 30 days). For the short-term intervention subgroup, the pooled RR was 0.65 (95% CI: 0.53–0.79), demonstrating a 35% reduction in recurrent ischemic stroke risk with DAPT compared to monotherapy. This greater percentage reduction highlights the stronger protective effect of DAPT in the short-term setting. Heterogeneity in this subgroup was relatively low (I^2^ = 27%; p = 0.18), indicating consistent results across studies. For the long-term intervention subgroup, the pooled RR was 0.72 (95% CI: 0.60–0.86), showing a 28% reduction in recurrent ischemic stroke risk with DAPT (Supplementary Figure S2). While still significant, the reduction was less pronounced compared to the short-term subgroup. Substantial heterogeneity was observed in this subgroup (I^2^ = 53%; p < 0.01), suggesting variability in the effects across studies.

#### Subgroup analysis for recurrent ischemic stroke by stroke type

Supplementary Figure S3 illustrates the findings of subgroup analysis (n = 28) evaluating the effectiveness of DAPT compared to any single antiplatelet therapy in preventing recurrent ischemic events, stratified by stroke type: ischemic stroke, lacunar stroke, and TIA or ischemic stroke. For ischemic stroke, the pooled RR was 0.58 (95% CI: 0.38–0.88), indicating a 42% reduction in recurrent ischemic stroke risk with DAPT compared to monotherapy. However, substantial heterogeneity was observed (I^2^ = 59%, p < 0.01), suggesting variability in the effects across studies. For lacunar stroke, the pooled RR was 0.64 (95% CI: 0.22–1.84), showing a 36% reduction in recurrent stroke risk, though the confidence interval includes 1, indicating uncertainty. Heterogeneity was moderate (I^2^ = 53%, p = 0.12). For TIA or ischemic stroke, the pooled RR was 0.75 (95% CI: 0.66–0.84), demonstrating a 25% reduction in recurrent ischemic events with DAPT (Supplementary Figure S3). Heterogeneity in this subgroup was lower (I2 = 38%, p = 0.07), indicating more consistent results across studies. The test for subgroup differences was not significant (p = 0.37), suggesting that the type of stroke did not significantly influence the overall effect of DAPT compared to monotherapy.

#### Subgroup analysis for recurrent ischemic stroke by DAPT regimen combinations

This subgroup analysis (Supplementary Figure S4) of recurrent ischemic stroke risk reveals that the choice of DAPT regimen significantly influences patient outcomes. While aspirin plus cilostazol showed a trend towards reducing stroke risk, this was not statistically significant (RR 0.52, 95% CI 0.19–1.39). However, the combination of aspirin and clopidogrel demonstrated a significant reduction in recurrent stroke (RR 0.69, 95% CI 0.59–0.82), aligning with its widespread use in clinical practice. In contrast, aspirin plus dipyridamole did not have a significant effect on recurrent stroke (RR 0.82, 95% CI 0.43–1.58). Interestingly, aspirin plus ticagrelor indicated a potential benefit (RR 0.80, 95% CI 0.68–0.93), suggesting this combination may warrant further investigation. Similarly, clopidogrel plus cilostazol also showed a significant reduction in recurrent stroke risk (RR 0.44, 95% CI 0.15–1.25), although the results were statistically non-significant. Importantly, the analysis revealed significant heterogeneity across the different DAPT regimens (I^2^ = 47%, p < 0.01), and a test for subgroup differences was also significant (p < 0.01) as shown in Supplementary Figure S4.

## Overall pooled effect of DAPT versus monotherapy on major adverse cardiovascular events (MACE)

**FIGURE 3:**
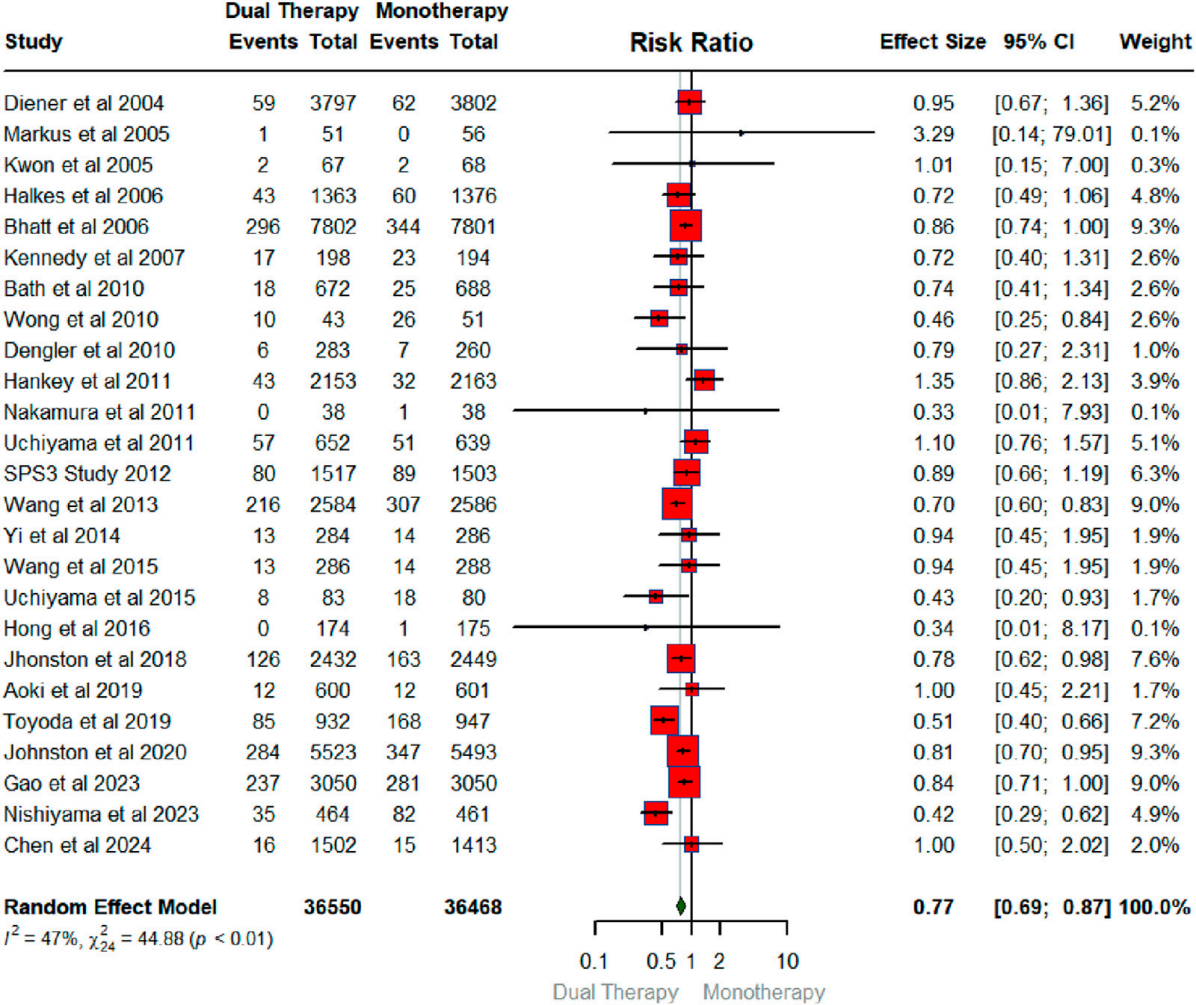
Forest plot summarizing the pooled effect of DAPT vs. monotherapy on MACE.

Figure 3 illustrates the pooled effect of DAPT versus any single antiplatelet therapy on MACE. The number of RCTs that contributed to MACE was 25 in total conducted between 2004 and 2024 with a sample size of 36,550 for intervention (DAPT therapy) and 36,468 for the control group (any single antiplatelet therapy). The findings showed that DAPT significantly reduced the risk of MACE by 23% (RR: 0.77; 95% CIs:0.69, 0.87). The heterogeneity statistics (I^2^ = 47%, X^2^: 44.88; P < 0.01) suggest small between-study variability.

### Subgroup analysis for MACE

#### Subgroup analysis for MACE by timing of DAPT initiation

The subgroup analysis of MACE by timing of DAPT initiation (Supplementary Figure S5) reveals a compelling trend: earlier initiation appears to be associated with a greater reduction in MACE. Notably, initiating DAPT within 12 h shows a trend towards reducing MACE (RR 0.78, 95% CI 0.62–0.98), approaching statistical significance. Furthermore, when DAPT is initiated within 24 h, the reduction in MACE becomes statistically significant (RR 0.81, 95% CI 0.72–0.93). This highlights the potential importance of early DAPT intervention in mitigating adverse cardiovascular events. In contrast, initiating DAPT at later time points, such as within 48 h or 72 h, does not demonstrate a statistically significant effect on MACE. Similarly, initiating DAPT between 7 and 180 days did not show a statistically significant reduction (RR 0.49, 95% CI 0.16–1.49) in MACE (Supplementary Figure S5). Overall, there is moderate heterogeneity across the subgroups (I^2^ = 48%), and the test for subgroup differences is significant (p < 0.01), reinforcing the notion that the timing of DAPT initiation plays a crucial role in influencing MACE outcomes.

#### Subgroup analysis for MACE by DAPT duration

The subgroup analysis explored the impact of DAPT duration on MACE (Supplementary Figure S6). Interestingly, both long-term DAPT (n = 25,220) and short-term DAPT (n = 11,330) showed a statistically significant reduction in MACE, with a relative risk of 0.77 (95% CI 0.65–0.92) for long-term and 0.77 (95% CI 0.69–0.85) for short-term. While there was significant heterogeneity within the long-term studies (I^2^ = 63%, p < 0.01), the short-term studies showed no heterogeneity (I^2^ = 0%, p = 0.63). Overall, the analysis supports the benefit of DAPT in reducing MACE, regardless of the duration studied, with no significant difference detected between the long-term and short-term subgroups (p = 0.92) as shown in Supplementary Figure S6.

#### Subgroup analysis for MACE by stroke type

Supplementary Figure S7 presents a subgroup analysis of the pooled effect of DAPT versus monotherapy on MACE, stratified by stroke type: ischemic stroke, lacunar stroke, and TIA or ischemic stroke. For ischemic stroke, DAPT demonstrated a trend towards reducing MACE (RR 0.75, 95% CI 0.58–0.95), with moderate heterogeneity observed (I^2^ = 42%, p = 0.07). In the lacunar stroke subgroup, DAPT showed a statistically non-significant reduction in MACE (RR 0.62, 95% CI 0.01–69.06), with extremely high heterogeneity (I^2^ = 89%, p < 0.01). For TIA or ischemic stroke, DAPT significantly reduced MACE (RR 0.81, 95% CI 0.73–0.90), with low heterogeneity (I^2^ = 22%, p = 0.23) as illustrated in Supplementary Figure S7. The test for subgroup differences was not significant (p = 0.63), suggesting that the effect of DAPT on MACE did not significantly differ across the stroke subtypes analyzed.

#### Subgroup analysis for MACE by DAPT regimen combinations

Supplementary Figure S8 presents a subgroup analysis of the pooled effect of DAPT on MACE, stratified by DAPT regimen combinations. This subgroup analysis of DAPT regimens for MACE indicated that aspirin plus clopidogrel (RR 0.82, 95% CI 0.75–0.91) and aspirin plus ticagrelor (RR 0.81, 95% CI 0.70–0.95) significantly reduced MACE. Aspirin plus cilostazol (RR 0.66, 95% CI 0.30–1.46) and clopidogrel plus cilostazol (RR 0.49, 95% CI 0.16–1.49) showed trends towards reducing MACE, but these were not statistically significant. Aspirin plus dipyridamole did not show a significant effect (RR 0.87, 95% CI 0.60–1.25). There was significant heterogeneity across the different DAPT regimens (I^2^ = 47%, p < 0.01), and a significant test for subgroup differences (p < 0.01) as shown in Supplementary Figure S8, highlighting that the choice of DAPT regimen significantly impacts MACE outcomes.

## Overall pooled effect of DAPT versus monotherapy on major bleeding

**FIGURE 4:**
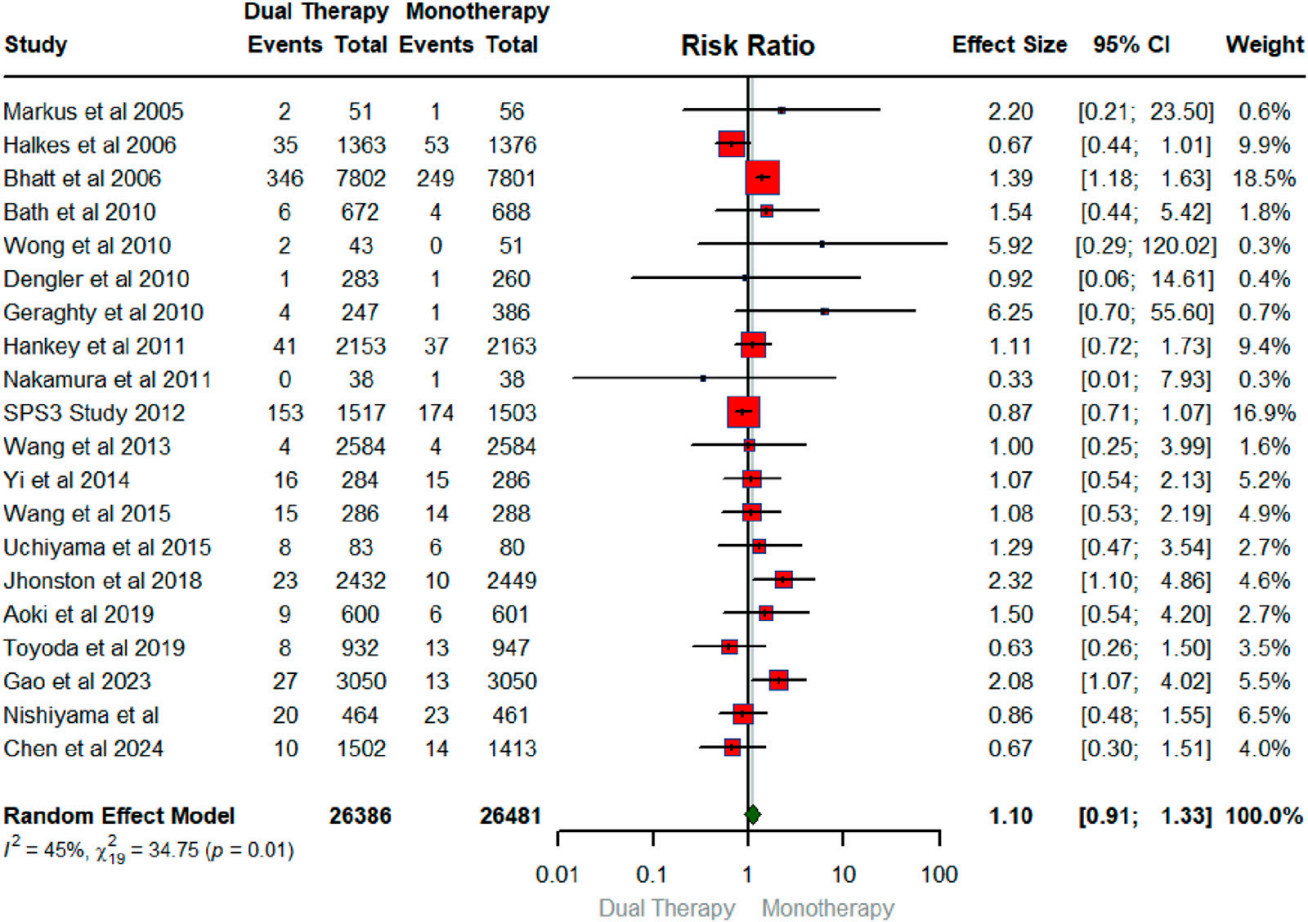
Forest plot summarizing the pooled effect of DAPT vs. monotherapy on major bleeding.

Figure 4 below exhibits the forest plot portraying the effect of DAPT on major bleeding. The number of RCTs that contributed to the major bleeding was 20 in total conducted between 2005 and 2024 with a sample size of 26,386 for intervention and 26,481 for the control group. The findings showed that DAPT did not have a significant effect on major bleeding (RR: 1.10; 95% CIs (0.91, 1.33). As shown in Figure 4, the heterogeneity statistics (I^2^ = 45%, X^2^:34.75; p-0.01) suggest low heterogeneity across the studies indicating a low between-study variability.

### Subgroup analysis for major bleeding

#### Subgroup analysis for major bleeding by timing of DAPT initiation

Supplementary Figure S9 presents a subgroup analysis of the pooled effect of DAPT on major bleeding, stratified by the timing of DAPT initiation. Initiating DAPT within 12 (RR 2.32, 95% CI 1.10–4.86) and 24 h (RR 1.34, 95% CI 1.20–1.49) significantly increased the risk of major bleeding. However, initiating DAPT within 48 h (RR 0.96, 95% CI 0.52–1.78) and between 7 and 180 days (RR 0.78, 95% CI 0.12–5.21) showed a non-significant effect on major bleeding, with no significant heterogeneity (I^2^ = 0%). Initiating DAPT within 72 h showed a non-significant effect on major bleeding (RR 1.30, 95% CI 0.38–4.45), but with significant heterogeneity (I^2^ = 70%, p = 0.02). The test for subgroup differences was significant (p < 0.01), indicating that the timing of DAPT initiation significantly impacts the risk of major bleeding.

#### Subgroup analysis for major bleeding by DAPT duration

Supplementary Figure S10 presents a subgroup analysis of major bleeding events comparing DAPT to monotherapy, stratified by study duration (long-term vs short-term). In the long-term DAPT subgroup (n = 20,753 intervention, n = 20,942 control), there was a increase in major bleeding events with DAPT (RR 1.13, 95% CI 0.84–1.53), thought with statistically non-significant results and with substantial heterogeneity (I^2^ = 65%, p < 0.01). In the short-term DAPT subgroup (n = 5,633 intervention, n = 5,539 control), there was no statistically significant difference in major bleeding events between DAPT and monotherapy (RR 1.06, 95% CI 0.78–1.43), with no significant heterogeneity (I^2^ = 0%, p = 0.86) as shown in Supplementary Figure S10. The test for subgroup differences was not significant (p = 0.70), indicating no significant difference in major bleeding risk between long-term and short-term DAPT.

#### Subgroup analysis for major bleeding by stroke type

This subgroup analysis (Supplementary Figure S11) examined the influence of stroke type on major bleeding risk with DAPT compared to monotherapy. For ischemic stroke (RR 0.99, 95% CI 0.74–1.32) and lacunar stroke (RR 0.87, 95% CI 0.84–0.90), DAPT did not significantly affect major bleeding risk. However, in the TIA or ischemic stroke subgroup, DAPT appears to be associated with an increase in major bleeding (RR 1.34, 95% CI 0.92–1.94), though with statistically non-significant results (Supplementary Figure S11). This subgroup difference (p = 0.02) suggests that the risk of major bleeding with DAPT may vary depending on the specific type of stroke.

#### Subgroup analysis for major bleeding by DAPT regimen combinations

This subgroup analysis evaluated the impact of different DAPT regimens on major bleeding events (Supplementary Figure S12). Interestingly, none of the DAPT combinations showed a statistically significant effect on major bleeding. Specifically, aspirin plus cilostazol (RR 1.29, 95% CI 0.49–3.40), aspirin plus clopidogrel (RR 1.23, 95% CI 0.95–1.59), aspirin plus dipyridamole (RR 0.73, 95% CI 0.34–1.56), and clopidogrel plus cilostazol (RR 0.78, 95% CI 0.12–5.21) all had confidence intervals that crossed 1, indicating no significant difference in bleeding risk compared to monotherapy. However, there was significant heterogeneity observed in the aspirin plus clopidogrel subgroup (I^2^ = 54%, p = 0.01), suggesting variability in bleeding risk within this group. Furthermore, the test for subgroup differences was significant (p = 0.02), indicating that the risk of major bleeding may vary across different DAPT combinations.

## Overall pooled effect of DAPT on hemorrhagic stroke

**FIGURE 5:**
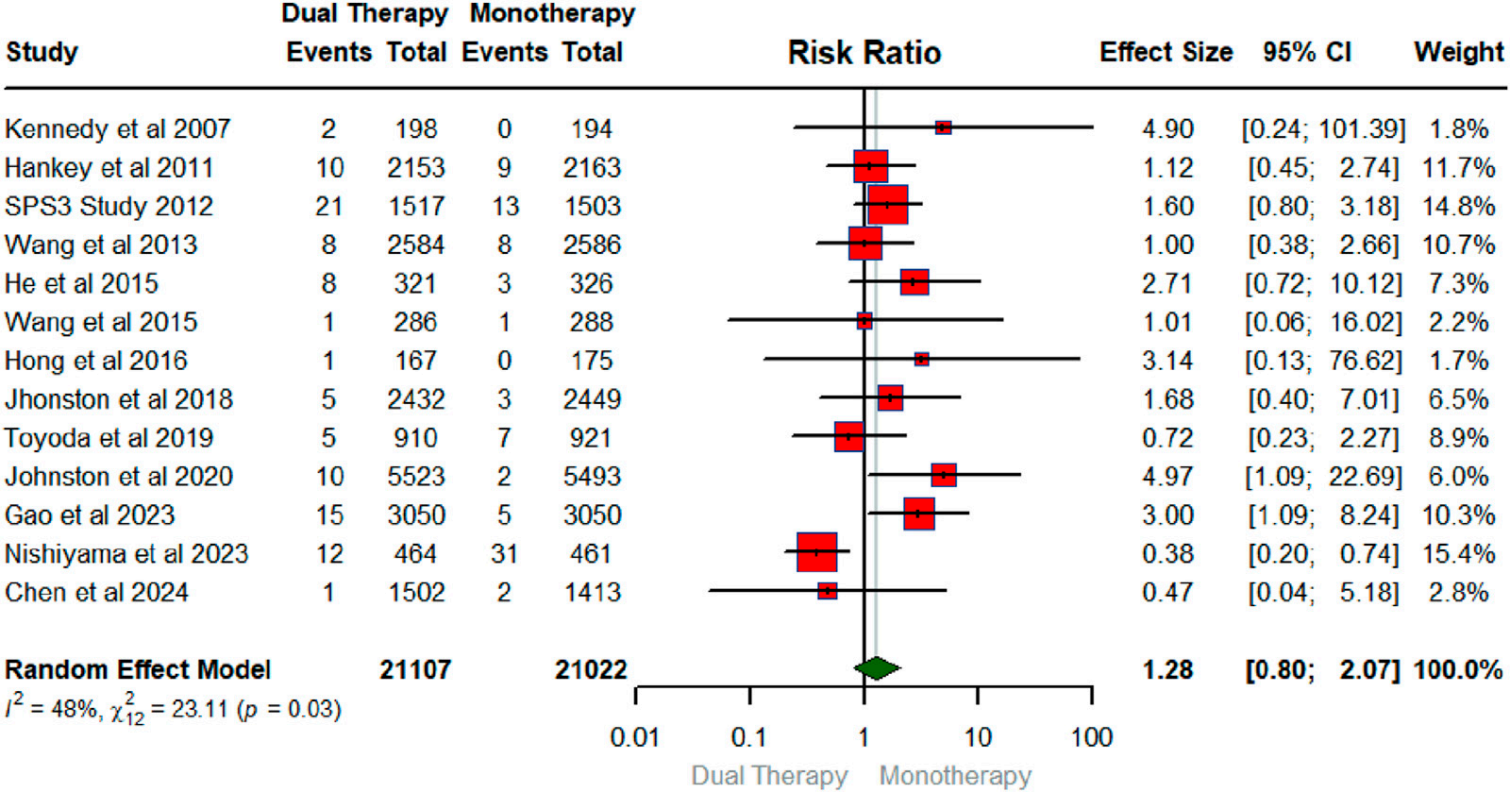
Forest plot summarizing the pooled effect of DAPT on hemorrhagic stroke.

Figure 5 displays the forest plot illustrating the pooled effect of DAPT versus any single antiplatelet therapy on hemorrhagic stroke. The number of RCTs that contributed to the hemorrhagic stroke was 13 in total conducted between 2007 and 2024 with a sample size of 21,107 for intervention and 21,022 for the control group. Compared to monotherapy, DAPT did not have a significant effect on hemorrhagic stroke (RR: 1.28; 95% CIs (0.80, 2.07). The heterogeneity statistics (I^2^ = 48%, X^2^: 23.11; p:0.03), suggest that between-study variability was low for the RCTs examining hemorrhagic stroke.

## Overall pooled effect of DAPT on mortality

**FIGURE 6:**
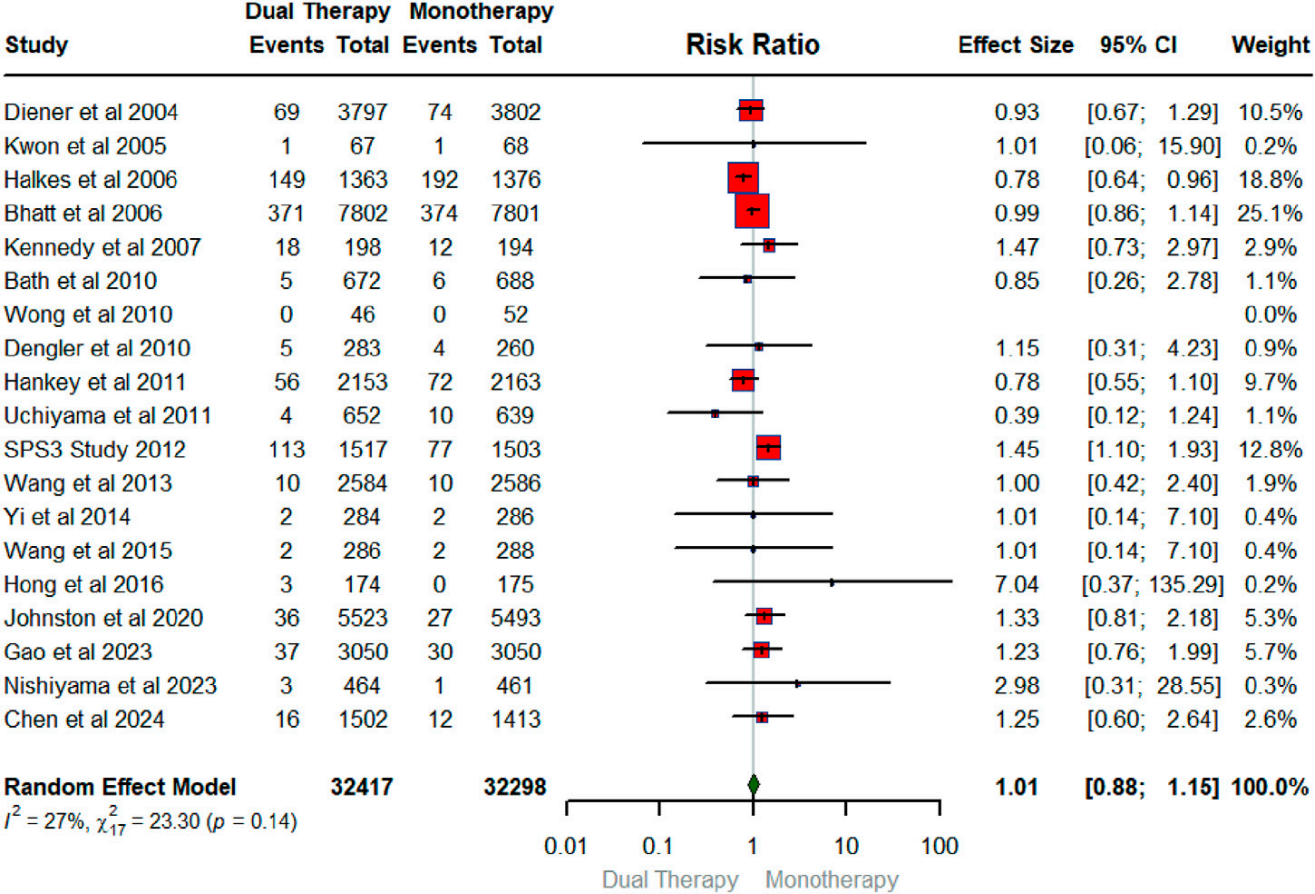
Forest plot summarizing the pooled effect of DAPT on mortality.

Figure 6 below exhibits the forest plot illustrating the pooled effect of DAPT versus any single antiplatelet therapy on Death. The number of RCTs that measured mortality as an outcome was 18 in total conducted between 2004 and 2024 with a sample size of 32,417 for intervention and 32, 298 for the control group. The findings showed that DAPT had no significant effect on mortality (RR: 1.01; 95% CIs: 0.88, 1.15). The heterogeneity statistics (I^2^ = 27%, X^2^:23.30; p:0.14) indicate low heterogeneity across the studies with, suggesting very small between-study variability.

## Findings of publication bias

**FIGURE 7:**
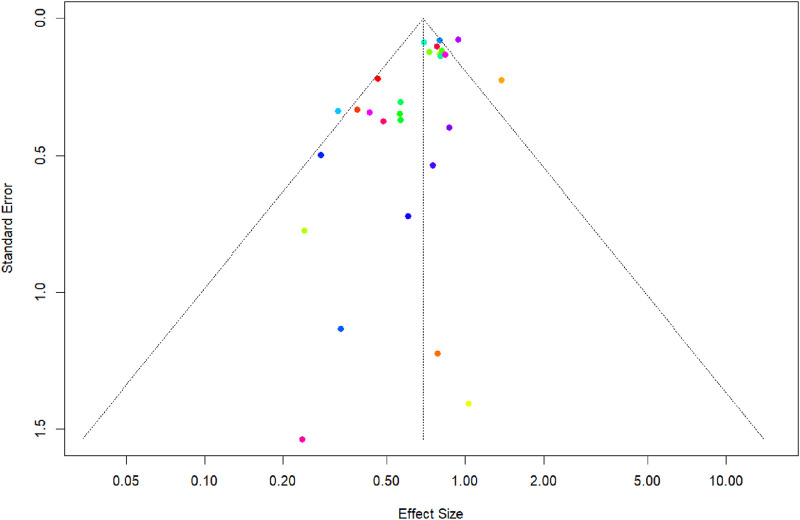
Funnel Plot and Egger’s regression test to assess the publication bias for recurrent ischemic stroke.

The funnel plot (Figure 7) assesses the potential for publication bias in a meta-analysis of DAPT versus monotherapy for primary outcome. Visual inspection of the funnel plot, which plots the standard error of each study against its effect size, suggests asymmetry. This is supported by a statistically significant result from Egger’s linear regression test for funnel plot asymmetry (t = −3.41, df = 26, p = 0.0021). The test indicates a significant negative association between standard error and effect size, with a bias estimate of −1.1983 (standard error = 0.3512). This suggests that smaller studies (with larger standard errors) are more likely to report larger effect sizes favoring DAPT, a pattern indicative of potential publication bias where smaller, less precise studies with null or negative findings may be underreported or unpublished.

## Discussion

This meta-analysis of 30 RCTs provides compelling evidence supporting the efficacy of DAPT compared to any single antiplatelet therapy in reducing the risk of recurrent ischemic stroke. The findings of the review and meta-analysis suggest that DAPT reduces the risk of recurrent ischemic stroke and cardiovascular outcomes. However, there is uncertainty about the role of dual antiplatelet therapy in reducing the risk of hemorrhagic stroke, major bleeding, and mortality. In fact, the direction of pooled risk ratio indicates that dual antiplatelet therapy may increase the risk of hemorrhagic stroke, however, the results are not statistically significant. Our analysis extends these findings by synthesizing a broader range of evidence and exploring the impact of key factors such as timing of intervention onset, stroke type, and treatment duration.

The mechanisms by which DAPT reduces the risk of recurrent ischemic stroke are multifactorial. DAPT, by combining two antiplatelet agents with different mechanisms of action, provides more comprehensive platelet inhibition compared to monotherapy (Brown et al., 2021). This synergistic effect reduces the likelihood of platelet activation and aggregation, key processes involved in thrombus formation and subsequent ischemic events (Chen et al., 2015). Specifically, aspirin inhibits cyclooxygenase (COX)-1, thereby reducing thromboxane A2 production, a potent vasoconstrictor and platelet activator (Simeone et al., 2022). Clopidogrel, ticagrelor, and other antiplatelet agents in the same class, inhibit the P2Y12 receptor on platelets, which is crucial for platelet aggregation and activation (Galli et al., 2024). The combination of these two mechanisms provides a more robust approach to preventing thrombus formation.

A key strength of our study lies in the detailed subgroup analyses, which offer valuable insights into the optimal utilization of DAPT, which offer valuable insights into the optimal utilization of DAPT. Our findings indicate that the benefit of DAPT is most pronounced, especially for outcomes such as recurrent ischemic stroke and MACE, when initiated early, specifically within 12 and 24 h of symptom onset. While a trend toward risk reduction was observed up to 72 h, the confidence intervals widened, suggesting greater uncertainty in the effect size. This observation underscores the importance of prompt DAPT administration following a stroke, aligning with the concept of “time is brain” (Gomez, 2018). The diminished effect observed with later initiation (7–180 days) warrants further investigation, though the wide confidence interval in this subgroup limits definitive conclusions. This finding may reflect the influence of other factors, such as stroke etiology or patient characteristics, on treatment response at later time points. Hence, the benefits of DAPT versus any single antiplatelet therapy with aspirin need to be interpreted cautiously after considering differences in the duration between randomization to DAPT and stroke onset.

However, our study finding suggested that Early DAPT (12–24 h) may increase bleeding risk, potentially due to disrupted hemostasis in the acute phase following stroke, especially when intracranial hemorrhage risk is elevated. In addition, the evidence suggests that patient characteristics like advanced age, low body weight, or comorbidities could further increase vulnerability (Khan et al., 2021). Careful patient selection and individualized risk assessment are crucial, particularly for those with higher baseline bleeding risks. Further research should identify which patients benefit most from early DAPT while minimizing bleeding.

Our analysis also explored the impact of DAPT duration, revealing that short-term DAPT (up to 30 days) appears to be associated with a greater reduction in recurrent ischemic events (compared to long-term DAPT (beyond 30 days). This observation raises the possibility that the maximal benefit of DAPT for secondary stroke prevention may be achieved with a shorter course of therapy. Upon critical analysis of the RCTs, it was found that the efficacy and safety of DAPT may not be found after the first 3 weeks of treatment onset. For example, findings from CHANCE and POINT trials suggest that DAPT versus monotherapy substantially reduces the risk of stroke for an initial 10 days to 2 weeks and the favorable effect of both DAPT and monotherapy appears to be the same after 10 days (Wang and Zhao, 2013; Johnston et al., 2018). This failure of DAPT to provide benefits after the first 3 weeks of treatment initiation is endorsed by a large SPS3 trial, a large multicenter trial with 3,020 patients (Investigators et al., 2012). More precisely, the findings of SPS3 trial documented that patient failed to benefit from dual antiplatelet therapy beyond the first 3 weeks of treatment onset. In fact, DAPT was found to be detrimental if the patients continued the DAPT beyond 3 weeks as it increased the risk of adverse events (Investigators et al., 2012). This finding contrasts somewhat with current clinical practice guidelines that often recommend long-term DAPT for secondary prevention (Chan et al., 2024), highlighting the need for further research to determine the optimal duration of DAPT and to explore the potential for tailoring treatment duration based on individual patient risk profiles. It is important to acknowledge that the definitions of short-term and long-term DAPT can vary across studies and clinical practice, and future research should aim to standardize these definitions to facilitate comparisons and improve clinical decision-making.

Lastly, our subgroup analyses revealed that the combination of aspirin and clopidogrel was particularly effective in reducing both recurrent stroke and MACE. This finding aligns with the widespread use of this DAPT regimen in clinical practice (Tan et al., 2015; Zhang et al., 2015; Paciaroni et al., 2019) and is likely influenced by the substantial number of studies investigating this combination, contributing to a larger sample size and increased statistical power. However, it is important to acknowledge that other DAPT combinations may also offer significant benefits but have not been as extensively studied. Therefore, we recommend conducting more RCTs to assess the efficacy and safety of alternative DAPT combinations, such as aspirin plus ticagrelor or clopidogrel plus cilostazol. This will provide a more comprehensive understanding of the comparative effectiveness of various DAPT regimens and allow for more personalized treatment strategies based on individual patient characteristics and risk profiles.

While the current review did not find a significant decrease in risk of hemorrhagic stroke and mortality with DAPT, the pooled risk ratio of greater than 1 suggests that DAPT may increase the risk of hemorrhagic stroke, though with statistically non-significant findings. As DAPT may decrease the risk of ischemic stroke and cardiovascular events, this could lead to a decreased mortality rate among these patients. Nonetheless, studies reporting on mortality as an outcome may have confounding factors such as patient’s age, gender, lifestyle, and socioeconomic status that could impact the true effects of DAPT on mortality. However, these findings are in agreement with existing evidence suggesting that long term DAPT versus monotherapy may not necessarily be favorable for reducing the risk of vascular events and mortality (Lee et al., 2013).

## Strengths and limitations

Our meta-analysis boasts several significant strengths that enhance its credibility and value. First, we conducted an extensive subgroup analysis that stratified data based on stroke type (ischemic stroke, lacunar stroke, and TIA or ischemic stroke), timing of intervention onset (within 12 h, 24 h, 48 h, 72 h, and 7–180 days), and duration of DAPT (short-term: up to 30 days; long-term: beyond 30 days). This approach allowed for a more nuanced understanding of DAPT’s effectiveness across various clinical contexts, providing robust evidence for the optimization of treatment strategies.

Despite these strengths, several limitations of the current meta-analysis need to be considered. First, a meta-analysis is a technique that may be constrained by publication bias, methodological rigor, and the comprehensiveness of the search strategy. Second, limitations of the original trials may influence the pooled results of the meta-analysis. Third, the duration of the trials and follow-up times were not consistent across the RCTs included in this study, which may explain, in part, the heterogeneity across the studies. Forth, the findings may not be generalizable to patients with varying risk profiles, such as those with moderate to severe strokes, cardiometabolic strokes, and patients receiving additional anticoagulation or fibrinolytic drugs. Finally, a limitation of our meta-analysis is the inability to perform a detailed subgroup analysis based on the TOAST classification of stroke subtypes. The included RCTs did not consistently report detailed information on the specific TOAST categories (Large-artery atherosclerosis, Cardioembolism, Small-vessel occlusion, Stroke of other determined cause, and Stroke of undetermined cause). Consequently, we performed subgroup analyses using the available data, which primarily categorized strokes as ischemic, lacunar, or TIA. Future research should prioritize the reporting of stroke subtypes according to the TOAST criteria to facilitate more precise meta-analyses and a deeper understanding of the effects of DAPT on different stroke etiologies.

Despite these limitations, our study provides valuable insights into the optimal use of DAPT in secondary stroke prevention. Future research should focus on conducting larger RCTs to directly compare different DAPT regimens and durations, identifying biomarkers to predict response to DAPT, and investigating the role of DAPT in specific stroke subtypes. These findings have important implications for clinical practice and highlight the need for individualized treatment strategies based on stroke type, timing of intervention, and patient-specific factors.

## Conclusion

Our findings affirm that DAPT reduces the risk of recurrent ischemic stroke and cardiovascular events. Nevertheless, there remains uncertainty regarding the role of DAPT in reducing the risk of hemorrhagic stroke, major bleeding episodes, and mortality. Given the high risk of recurrent stroke after a transient ischemic attack or ischemic stroke, a short course of DAPT may be beneficial. The timing of initiating DAPT is crucial, with the most favorable effects for recurrent ischemic stroke and MACE observed when treatment is started within 12–24 h post-stroke. The short-term use of DAPT (up to 30 days) was associated with a greater percentage reduction in recurrent ischemic stroke risk compared to long-term use, highlighting its stronger protective effect in the acute phase. This consistency in the short-term subgroup supports the efficacy of DAPT in preventing recurrent ischemic events during the critical early period following a stroke or TIA. We also found that aspirin plus clopidogrel is a good combination in reducing the risk of recurrent ischemic stroke and MACE. However, clinicians should carefully consider when and how to use DAPT, as it is not a one-size-fits-all solution. More research is needed to fine-tune the best ways to use DAPT for different types of strokes and patients.

## Clinical implications and future directions

The findings underscore the potential of DAPT in reducing the risk of recurrent ischemic events, emphasizing the importance of early intervention. Given the high risk of recurrent stroke after a TIA or ischemic stroke, a short course of DAPT may be particularly beneficial. However, prolonged use of DAPT may increase the risk of hemorrhage or major bleeding compared to monotherapy, necessitating careful consideration of the risks and benefits based on the patient’s clinical profile. Individualized treatment strategies should be employed, taking into account stroke type, timing of intervention, and patient-specific factors to optimize outcomes. Future research should focus on conducting larger RCTs to directly compare different DAPT regimens and durations. Identifying biomarkers to predict response to DAPT and investigating the role of DAPT in specific stroke subtypes are also crucial areas for exploration. These efforts will provide a clearer understanding of the optimal use of DAPT in secondary stroke prevention and further refine clinical guidelines to enhance patient care.

## Data Availability

The original contributions presented in the study are included in the article/Supplementary Material, further inquiries can be directed to the corresponding author.
