# Supplementary material for: Tailoring dual antiplatelet therapy for stroke prevention: a meta-analysis of timing, duration, regimen, and stroke subtypes
Source: Front Pharmacol. 2025 Apr 24;16:1516402. doi: 10.3389/fphar.2025.1516402 (PMC12058503; doi:10.3389/fphar.2025.1516402)
Supplement: Supplementary file 1 [file Table1.docx]

| **Supplemental Table 1: Search Strategy: Embase/MEDLINE/Pubmed** | | | | |
| --- | --- | --- | --- | --- |
| **PICO Element** | **Search Terms (Paraphrased & Organized)** | **Databases** | **Date Range** | **Notes** |
| Population | * Stroke <br> * Ischemic Stroke <br> * Cerebral Infarction <br> * Cerebral Ischemia <br> * Transient Ischemic Attack <br> * Adults (including Young Adult, Middle Aged, Aged, Very Elderly) | PubMed, Web of Science, EMBASE | 2000 - December 2024 | Age limits were applied to all population terms. |
| Intervention | * Dual Antiplatelet Therapy <br> * Combined Antiplatelet Treatment <br> * Dual vs. Monoantiplatelet Therapy | PubMed, Web of Science, EMBASE | 2000 - December 2024 |  |
| Comparison | * Single Antiplatelet Agent <br> * Antiplatelet Agent (used in combination with other terms) | PubMed, Web of Science, EMBASE | 2000 - December 2024 |  |
| Outcomes | * Recurrent Ischemic Stroke <br> * Hemorrhagic Stroke <br> * Cardiovascular Events <br> * Major Bleeding <br> * Mortality | PubMed, Web of Science, EMBASE | 2000 - December 2024 |  |
| Study Design | * Randomized Controlled Trial | PubMed, Web of Science, EMBASE | 2000 - December 2024 | This term was used to filter for RCTs. |
| Specific Antiplatelet Agents (for broader search) | * Aspirin <br> * Clopidogrel <br> * Ticagrelor <br> * Dipyridamole <br> * Cilostazol | PubMed, Web of Science, EMBASE | 2000 - December 2024 | These terms were searched individually to capture all relevant studies regardless of combination. |
