# Supplementary material for: Tailoring dual antiplatelet therapy for stroke prevention: a meta-analysis of timing, duration, regimen, and stroke subtypes
Source: Front Pharmacol. 2025 Apr 24;16:1516402. doi: 10.3389/fphar.2025.1516402 (PMC12058503; doi:10.3389/fphar.2025.1516402)
Supplement: Supplementary file 2 [file Table2.docx]

| **Supplemental Table 2: Definition of bleeding as defined by authors in respective RCTs** | | |
| --- | --- | --- |
| **Study** | **Trial Name** | **Definition of Bleeding** |
| Diener et al 2004 [14] | MATCH | Life-threatening bleeding is characterized by any fatal hemorrhagic event, a decrease in hemoglobin of at least 5 g/L, severe hypotension requiring inotropic support (hemorrhagic shock), symptomatic intracranial hemorrhage, or the necessity for transfusion of at least four units of red blood cells or an equivalent volume of whole blood. Major bleeding is defined as a condition causing substantial long-term impairment, intraocular hemorrhage resulting in significant vision loss, or the requirement for transfusion of at least three units of red blood cells or an equivalent volume of whole blood. |
| Markus et al 2005 [15] | CARES | Bleeding events were categorized as "life-threatening," "major (e.g., intracranial bleeding)," and "minor (e.g. epistaxis)," but clear definitions for major and life-threatening bleeding were not provided. For instance, epistaxis was included, but its classification within these categories was not explicitly clarified. |
| Halkes et al 2006 [17] | ESPRIT | The outcome of major bleeding complications encompassed all intracranial hemorrhages, any fatal bleeding event, or any bleeding necessitating hospital admission. Major bleeding complications were further classified into non-fatal extracranial bleeding, fatal extracranial bleeding, non-fatal intracranial bleeding, and fatal intracranial bleeding. |
| Bhatt et al 2006 [18] | CHARISMA | Severe bleeding was as defined by the Global Utilization of Streptokinase and Tissue Plasminogen Activator for Occluded Coronary Arteries (GUSTO) criteria. This included fatal hemorrhage, intracranial bleeding, or bleeding that resulted in hemodynamic instability necessitating blood or fluid resuscitation, inotropic support, or surgical intervention. Additionally, moderate bleeding, as classified by the GUSTO criteria—requiring transfusion but not meeting the threshold for severe bleeding—was assessed, along with fatal bleeding and primary intracranial hemorrhage. |
| Kennedy et al 2007 [19] | FASTER | Hemorrhagic events were categorized and assigned into two main groups based on the source of bleeding: intracranial and extracranial. The extracranial group was further subdivided into the following categories:   1. **Severe**: Defined as life-threatening bleeding leading to hemodynamic instability or hypovolemic shock, necessitating inotropic support or other interventions to maintain cardiac output, requiring a transfusion of more than 2 units of packed red blood cells, or associated with a drop in hemoglobin levels of 5 g/L or greater. 2. **Moderate**: Defined as bleeding requiring a transfusion of 2 units of packed red blood cells or fewer, not meeting the criteria for severe bleeding, or associated with a hemoglobin drop of less than 5 g/L. 3. **Mild**: Defined as bleeding that does not require a transfusion, does not cause hemodynamic compromise, and typically includes conditions such as hematoma, subcutaneous bleeding, or oozing from puncture sites. Such cases may necessitate adjustments to the medication regimen. 4. **Asymptomatic**: Defined as bleeding that does not produce any noticeable symptoms. |
| Bath et al 2010 [21] | PROFESS | Major hemorrhagic events were classified into two categories: life-threatening and non-life-threatening. Hemorrhagic events, whether major or minor, were further categorized based on their type or location, including hematuria (blood in urine), hematemesis (vomiting blood), gastrointestinal bleeding, epistaxis (nosebleeds), intraocular bleeding (bleeding within the eye), purpura (bruising or bleeding under the skin), gynecologic bleeding, internal bleeding (bleeding within body cavities or organs), intracranial bleeding (bleeding within the skull), and other types of bleeding not fitting the above categories. This classification system was used to systematically identify and document the nature and severity of hemorrhagic events in the study. |
| Wong et al 2010 [22] | CLAIR | Hemorrhagic events occurring between day 1 and day 7, defined as any of the following: symptomatic hemorrhagic transformation of cerebral infarction, symptomatic intracerebral hemorrhage unrelated to cerebral infarction, serious extracranial hemorrhage (e.g., gastrointestinal bleeding, hematoma, or hematuria), death from any cause, or death due to hemorrhagic complications. Serious hemorrhage was defined as any symptomatic intracranial hemorrhage or any hemorrhage necessitating blood transfusion or extended hospitalization. |
| Dengler et al 2010[23] | EARLY | Not defined: Author had only mentioned major bleeding complication |
| Nakamura et al 2011[24] | NA | Not defined: Author had only mentioned major bleeding complication |
| Wang et al 2013[27] | CHANCE | A moderate-to-severe bleeding event was defined according to the Global Utilization of Streptokinase and Tissue Plasminogen Activator for Occluded Coronary Arteries (GUSTO) criteria. Severe hemorrhage included fatal bleeding, intracranial hemorrhage, or any hemorrhage causing hemodynamic instability that required blood or fluid replacement, inotropic support, or surgical intervention. Moderate hemorrhage was defined as bleeding that necessitated a blood transfusion but did not result in hemodynamic compromise requiring further intervention. |
| Yi et al 2014 [29] | NA | Hemorrhagic episodes were defined as hemorrhagic transformation of cerebral infarction, intracerebral hemorrhage unrelated to cerebral infarction, or extracranial hemorrhage (such as gastrointestinal bleeding or hematuria). Serious hemorrhage was classified as any symptomatic intracranial hemorrhage or any hemorrhage requiring blood transfusion or extended hospitalization. |
| He et al 2015 [30] | NA | Intracranial or extracranial hemorrhagic events |
| Hong et al 2016 [33] | COMPRESS | Bleeding events were categorized into three groups: life-threatening, major, or minor. Life-threatening bleeding included events such as death related to hemorrhagic complications, a hemoglobin drop greater than 5 g/dL, hypovolemic shock due to bleeding, symptomatic intracranial hemorrhage, or the need for a transfusion of 4 or more units of blood. Major bleeding was defined as intraocular hemorrhage, bleeding causing significant disability, or requiring a transfusion of 3 or fewer units of blood. Minor bleeding included all other bleeding events that did not meet the criteria for life-threatening or major bleeding. |
| Zuo et al 2017 [34] | NA | Several hemorrhagic events were monitored for safety purposes, following the Global Utilization of Streptokinase and Tissue Plasminogen Activator for Occluded Coronary Arteries (GUSTO) definition. These events included intracranial hemorrhage and gastrointestinal bleeding. Additionally, nasal and gum bleeding were also assessed as part of the safety evaluation. |
| Jhonston et al 2018 [35] | POINT | The risk of major hemorrhage was defined as symptomatic intracranial hemorrhage, intraocular bleeding leading to vision loss, transfusion of 2 or more units of red blood cells or an equivalent amount of whole blood, hospitalization or extension of an existing hospital stay, or death caused by hemorrhage. Secondary safety outcomes included hemorrhagic stroke, symptomatic intracerebral hemorrhage, other symptomatic intracranial hemorrhage, major hemorrhage outside the intracranial region, minor hemorrhage (including asymptomatic intracranial hemorrhage), and death from any cause. |
| Aoki et al 2019 [36] | NA | Intracerebral hemorrhage and subarachnoid hemorrhage occurring within 14 days of onset. Additionally, intracranial and extracranial hemorrhages were assessed both within 14 days and within 3 months of the initial event. |
| Johnston et al 2020 [38] | THALES | The primary safety outcome was the first severe bleeding event, defined as a composite of the first occurrence of intracranial hemorrhage or fatal bleeding, the first moderate or severe bleeding event, premature and permanent discontinuation of the trial treatment due to any bleeding |
| SPS3 Study 2012 [26] | SPS3 | Intracranial hemorrhages were categorized based on their location, including intracerebral, subdural, epidural, and subarachnoid hemorrhages, as confirmed by neuroimaging. |
| Gao et al 2023 [39] | INSPIRES | Moderate to severe bleeding, as defined by the GUSTO trial criteria, includes severe or life-threatening bleeding (such as intracranial hemorrhage or bleeding causing significant hemodynamic compromise requiring intervention) and moderate bleeding (which requires blood transfusion but does not cause hemodynamic instability). These criteria were used to assess bleeding risks in patients receiving thrombolytic therapies. |
| Chen et al 2024 [41] | ATAMIS | Bleeding was defined based on the Heidelberg bleeding classification, which categorizes bleeding events such as mucocutaneous hemorrhage, organ hemorrhage, and intracranial hemorrhage. These events were monitored and recorded within a 14-day timeframe. |
| Nishiyama et al [40] | CSPS.com Trial | Bleeding was defined according to the GUSTO classification, which includes severe or life-threatening bleeding such as symptomatic intracranial hemorrhage (e.g., hemorrhagic stroke, subdural or epidural hemorrhage) and bleeding causing significant hemodynamic compromise requiring treatment. |
| Uchiyama et al 2015 [32] | CATHARSIS | Bleeding was defined as symptomatic intracranial hemorrhage and major extracranial hemorrhage requiring hospitalization. These criteria focus on severe bleeding events that are clinically significant, either occurring within the brain (intracranial) or outside the brain (extracranial) and necessitating hospital care. |
| Kwon et al 2005 [16] | TOSS | Hemorrhoid or Nasal bleeding are mentioned but there is no proper definition of bleeding |
| Uchiyama et al 2011 [25] | JASAP | There is no proper definition of bleeding |
| Geraghty et al 2010 [20] | EXPRESS | There is no proper definition of bleeding |
| Han et al 2013 [28] | ECLIPSE | Not measured as outcome |
